# Active beating modes of two clamped filaments driven by molecular motors

**DOI:** 10.1098/rsif.2021.0693

**Published:** 2022-01-05

**Authors:** Laura Collesano, Isabella Guido, Ramin Golestanian, Andrej Vilfan

**Affiliations:** ^1^ Max Planck Institute for Dynamics and Self-Organization (MPIDS), Göttingen 37077, Germany; ^2^ Rudolf Peierls Centre for Theoretical Physics, University of Oxford, Oxford OX1 3PU, UK; ^3^ Jožef Stefan Institute, Ljubljana 1000, Slovenia

**Keywords:** artificial cilia, elastic filaments, molecular motors, buckling instability, microtubules, dynein

## Abstract

Biological cilia pump the surrounding fluid by asymmetric beating that is driven by dynein motors between sliding microtubule doublets. The complexity of biological cilia raises the question about minimal systems that can re-create similar patterns of motion. One such system consists of a pair of microtubules that are clamped at the proximal end. They interact through dynein motors that cover one of the filaments and pull against the other one. Here, we study theoretically the static shapes and the active dynamics of such a system. Using the theory of elastica, we analyse the shapes of two filaments of different lengths with clamped ends. Starting from equal lengths, we observe a transition similar to Euler buckling leading to a planar shape. When further increasing the length ratio, the system assumes a non-planar shape with spontaneously broken chiral symmetry after a secondary bifurcation and then transitions to planar again. The predicted curves agree with experimentally observed shapes of microtubule pairs. The dynamical system can have a stable fixed point, with either bent or straight filaments, or limit cycle oscillations. The latter match many properties of ciliary motility, demonstrating that a two-filament system can serve as a minimal actively beating model.

## 1. Introduction

Cilia and flagella are cellular appendages that can spontaneously beat in an asymmetric or undulatory fashion in order to transport the surrounding fluid or propel a swimming microorganism [1]. Altogether, several hundred different proteins are involved in maintaining the structural stability of the cilium, generating and controlling the beating, as well as supplying materials and energy [2]. The beating is powered by axonemal dynein motors that induce a shearing force between pairs of doublet microtubules. The control mechanism that activates the dynein motors and maintains the beating, however, is not yet well understood. An attractive hypothesis is that the dynein motors react to the sliding motion of the filaments with effective negative damping at a certain frequency [3,4]. There are several processes by which motor proteins can induce spontaneous oscillations [5]. An alternative proposition is the ‘geometric clutch’ model, which was initially based on the qualitative notion that a buckled filament loses contact with the motors, which thus become inactivated [6]. More recently, it has been proposed that the motors are controlled by transverse stress, which is coupled to the curvature in helically twisted axonemes [7].

The structural and biochemical complexity of a cilium and its elusive control mechanism lead to the question whether it is possible to design a minimal system that reproduces the spontaneous beating dynamics of cilia and flagella. The ability of tangential forces to produce undulatory motion has been discussed in several theoretical studies [8–14]. At high densities, such filaments self-organize into a rich collection of mesoscopic phases [15]. However, in these models, the filaments are propelled by external forces, rather than shear forces within the axoneme. Models based on internal forces between connected elastic filaments [16] can explain faster beating that is not limited by the speed of motors. The shearing between connected filaments also leads to the counterbend phenomenon—bending the axoneme in one direction induces an opposite bend some characteristic distance away, thus facilitating wave generation [17,18].

Early attempts to build biomimetic cilia have concentrated on magnetic [19–21] or electrostatic [22] actuation mechanisms to produce non-reciprocal beating. Periodic beating has also been achieved with bundles of microtubules interacting with clusters of kinesin-1 [23]. A minimal system that produces flagella-like beating has been created with a microtubule that is clamped to the surface with one end and pushed along its length by motors attached to the surface [24]. Nevertheless, experiments with motors attached to the surface differ in crucial aspects from biological cilia. The filaments are driven by external forces, rather than internal forces as in cilia. Filament velocity is largely limited by the velocity of the motors, whereas the velocity of a cilium tip can surpass that of the dynein motors by several orders of magnitude. The proximity to the surface also makes filaments unsuitable for generating fluid flows. Alternative mechanisms where the beating originates between filaments and without direct contact to the substrate are therefore of a high interest. We recently reported on the bottom-up assembly of a minimal synthetic axoneme (synthoneme) consisting of two microtubules, growing from a common seed, and a patch of self-assembled dynein motors on one of them [25]. When the patch is relatively short, the filaments beat in a discrete fashion, switching between a buckled and a straight state.

**Figure 1.**
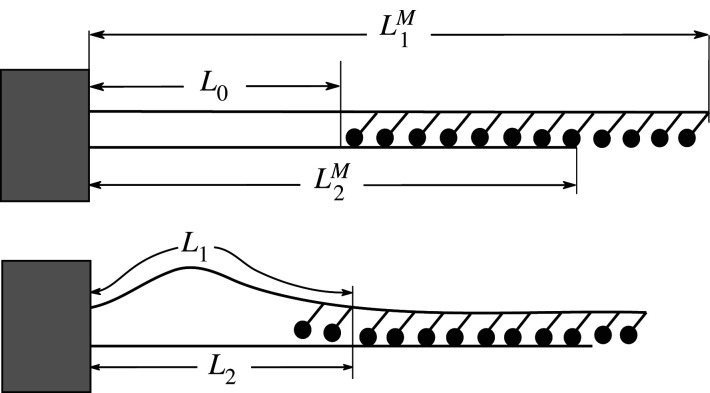
Schematic of the system of two elastic filaments and motor proteins exerting a force between them.

In this paper, we study theoretically a system with two filaments clamped on one end and interacting via molecular motors along their length (figure 1). We demonstrate that such systems are able to reproduce periodic beating of a cilium. Additionally, we show that under these conditions (or *in vitro*) a broader class of configurations of microtubule–motor protein systems is accessible. We describe the filaments as linear elastica. For the motors, we use a simple continuum model in which a longitudinal force slows the motors down according to a linear force–velocity relationship. A normal force, when exceeding a threshold value, leads to the detachment (unzipping) of motors. A related model in which only one of the two filaments is allowed to bend has been studied by Brokaw [26] to explain the dynamics in frayed natural axonemes [27]. Here, we provide a full range of solutions for the system of two clamped filaments and a general discussion of the dynamic regimes induced by the molecular motors acting between them. We further compare the calculated static shapes to experimental images of coupled microtubules driven by dynein motors and obtain an excellent fit.

## 2. Static problem

Our solution is based on a timescale separation: the elastic filaments reach their quasi-static configuration quickly while the actuation by molecular motors takes place on a slower timescale. We therefore first determine the equilibrium configuration of two elastic filaments whose ends are clamped to one another. We parameterize each filament *i* = 1, 2 with the position vector **x**_*i*_(*s*) as a function of the arc length *s* ∈ [ − *L*_*i*_/2, *L*_*i*_/2] measured from its centre. The tangent vector is given by $t_{i}(s)=dx_{i}/ds$. The stress in each rod is described with the force **F**_*i*_ and the bending moment (torque) **M**_*i*_(*s*). We assume that the motors cannot exert a torsional torque on the filaments, therefore **M**_*i*_ · **t**_*i*_ = 0. The clamped ends impose the boundary conditions

2.1 $$x_{1}(\pm L_{1}/2)=x_{2}(\pm L_{2}/2)$$

and

2.2 $$t_{1}(\pm L_{1}/2)=t_{2}(\pm L_{2}/2).$$

In addition, the ends are force- and torque-free

2.3 $$F_{1}+F_{2}=0\;\mathrm{and}\;M_{1}(\pm L_{1}/2)+M_{2}(\pm L_{2}/2)=0.$$

Between the endpoints, the filaments obey the elastic rod equations [28]

2.4 $$\frac{dM_{i}}{ds}=F_{i}\times t_{i}\;\mathrm{and}\;M_{i}=EI\;t_{i}\times \frac{dt_{i}}{ds}.$$

### 2.1. Small amplitude limit

We first solve the problem in the limit of small deflections, which is the case for small length differences, *L*_1_ − *L*_2_ ≪ *L*_2_. Both filaments are then deformed in the same plane and can be parameterized with the tangent angles *ϕ*_*i*_(*s*) ≪ 1. The linearized beam equation reads

2.5 $$EI\frac{d^{2}\phi_{i}(s)}{ds^{2}}=F_{i}\phi_{i}(s).$$

For *F*_1_ = −*F* and *F*_2_ = *F* (*F* > 0), it can be solved using the ansatz

2.6 $$\phi_{1}(s)=A_{1}\sin(ks)\;\mathrm{and}\;\phi_{2}(s)=A_{2}\sinh(ks),$$

with $k=\sqrt{F/EI}$. The boundary conditions from equation (2.2) require *ϕ*_1_(*L*/2) = *ϕ*_2_(*L*/2) and from equation (2.3) *ϕ*_1_′(*L*/2) + *ϕ*_2_′(*L*/2) = 0. Together, they lead to the equation tanh (*kL*/2) = −tan(*kL*/2) with the lowest non-trivial solution *kL* = 4.730. The critical load for the buckling transition follows as:

2.7 $$F_{\mathrm{crit}}=2.267\frac{\pi^{2}EI}{L^{2}}.$$

The critical load of a filament clamped to another elastic filament therefore lies between the Euler critical load with pinned ends (*π*^2^*EI*/*L*^2^) and that with clamped ends (4*π*^2^*EI*/*L*^2^) [28,29]. This critical load allows us to estimate the total force needed from the molecular motors to leave the straight configuration, which is a prerequisite on the way to active beating.

### 2.2. Planar solution

Above the critical load, we solve the rod equation (2.4) numerically. Again, we use a parametrization starting from the midpoint of each filament and orient the system such that the solutions are symmetric with respect to the rotation around the *z*-axis. The symmetry implies $t_{1}⊥{\hat{e}}_{z}$ and $F⊥{\hat{e}}_{z}$.

We first determine the planar solutions of the two-filament system. In this case, each filament is still described with a single tangent angle *ϕ*_*i*_(*s*), but the nonlinear equations read *EIϕ*_*i*_″(*s*) = *F*_*i*_ sin(*ϕ*_*i*_(*s*)) or

2.8 $$\frac{EI}{2}{\left(\frac{d\phi_{i}}{ds}\right)}^{2}\mp F\cos\phi_{i}=C_{i},$$

where the negative sign applies for *i* = 1 and positive for *i* = 2. The filament shapes are determined by the differential equations

2.9 $$\frac{dx_{i}}{ds}=\cos\phi_{i}\;\mathrm{and}\;\frac{dz_{i}}{ds}=\sin\phi_{i}.$$

**Figure 2.**
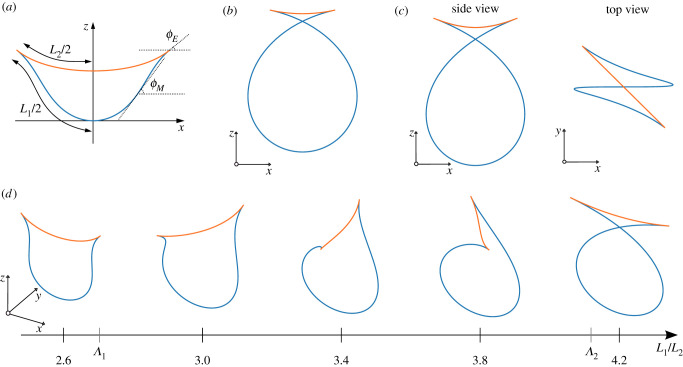
Planar (*a*,*b*) and 3D (*c*) solutions for the shape of two clamped filaments of lengths *L*_1_ and *L*_2_. (*a*) The first solution is characterized by an inflection point: along the arc length, *ϕ*_1_ changes from 0 to a maximum *ϕ*_*M*_ and then decreases to *ϕ*_*E*_. The length ratio is *L*_1_/*L*_2_ = 1.37. (*b*) In the second solution, *ϕ*_1_ starts at *π* and then falls monotonously to *ϕ*_*E*_ (shown for *L*_1_/*L*_2_ = 6.94). (*c*) At intermediate length ratios (here *L*_1_/*L*_2_ = 3.85), the stable solution assumes a non-planar shape with either left-handed (shown) or right-handed chirality. (*d*) Transition from planar to non-planar and back to planar shapes as the length ratio *L*_1_/*L*_2_ increases.

For given values of *F* and the integration constants *C*_*i*_, the solutions *s*(*ϕ*), *x*(*ϕ*) and *z*(*ϕ*) can be expressed with elliptic integrals. The symmetry of the solutions is *ϕ*( − *s*) = −*ϕ*(*s*) and the boundary conditions *x*_1_(*L*_1_/2) = *x*_2_(*L*_2_/2), *ϕ*_1_(*L*_1_/2) = *ϕ*_2_(*L*_2_/2) = *ϕ*_*E*_ and *C*_1_ + *F*cos*ϕ*_*E*_ = *C*_2_ − *F*cos*ϕ*_*E*_. The boundary conditions can be satisfied in two ways: (i) filament 1 has an inflection point, i.e. the curvature $d\phi_{1}/ds$ changes sign between *s* = 0 and *s* = *L*_1_/2 (figure 2*a*), or (ii) filament 1 starts with *ϕ*_1_(0) = *π* and has a negative derivative throughout the solution (figure 2*b*). Solutions with a larger number of inflection points are possible, but they are unstable, even in 2D confinement [30].

In case (i), which holds for small length differences, the two filament equations can be rewritten as

2.10 $$\frac{EI}{2F}{\left(\frac{d\phi_{1}}{ds}\right)}^{2}=\cos\phi_{1}-\cos\phi_{M}$$

and

2.11 $$\frac{EI}{2F}{\left(\frac{d\phi_{2}}{ds}\right)}^{2}=2\cos\phi_{E}-\cos\phi_{M}-\cos\phi_{2},$$

where *ϕ*_*M*_ is the angle at the inflection (figure 2*a*). Their solution in explicit form is given in electronic supplementary material, §A. The equations contain two non-trivial parameters (*ϕ*_*E*_ and *ϕ*_*M*_) and have to satisfy the conditions *L*_1_/*L*_2_ = *s*_1_(*ϕ*_*E*_)/*s*_2_(*ϕ*_*E*_) and *x*_1_(*ϕ*_*E*_) = *x*_2_(*ϕ*_*E*_). We find numerically that they are solvable for $0\leq \phi_{E}\leq \phi_{E}^{\max}=0.941$. The maximum *ϕ*_*E*_ corresponds to a solution with $L_{1}/L_{2}=\Lambda_{1}=2.70$.

Solution (ii) is valid for large *L*_1_/*L*_2_ ratios, for which filament 1 forms a loop. Equations for solution (ii) can be written as

2.12 $$\frac{EI}{2F}{\left(\frac{d\phi_{1}}{ds}\right)}^{2}=C-\cos\phi_{1}$$

and

2.13 $$\frac{EI}{2F}{\left(\frac{d\phi_{2}}{ds}\right)}^{2}\;=\;C-2\cos\phi_{E}+\cos\phi_{2}$$

and the solutions are a function of *C* and *ϕ*_*E*_ (see electronic supplementary material, §B for an explicit form). They can be found for 0 ≤ *ϕ*_*E*_ ≤ 0.255—the highest angle corresponds to $L_{1}/L_{2}=\Lambda_{2}=4.12$.

### 2.3. Non-planar solution

The discontinuity between simply bent and looped configurations suggests that a filament that is continuously pushed by the motors bends out-of-plane during the shape transition. It is known from the literature that a single rod with two clamped ends undergoes a secondary bifurcation beyond which the shape becomes non-planar [14,31,32]. Here, we analyse the possible 3D shapes of two clamped filaments numerically. To solve the 3D shape of two filaments, we first integrate both filament equations for a set of initial values **t**_1_(0), **t**_2_(0), **F**, **M**_1_(0) and **x**_2_(0) − **x**_1_(0). We then determine the parameters that fulfil the boundary conditions (2.1) and (2.2). If we fix **x**_1_(0) = 0 and $t_{1}(0)={\hat{e}}_{x}$, we get a system with five independent variables ($t_{2}(0)\cdot {\hat{e}}_{x}$, $x_{2}(0)\cdot {\hat{e}}_{z}$, *F*_*x*_, *F*_*y*_ and *M*_1*y*_) that has to satisfy five equations (three components of **x**_1_(*L*_1_/2) = **x**_2_(*L*_2_/2), two independent components of **t**_1_(*L*_1_/2) = **t**_2_(*L*_2_/2)). In the range $\Lambda_{1}<L_{1}/L_{2}<\Lambda_{2}$, there is a set of non-planar solutions that spontaneously break the chiral symmetry. They represent configurations with minimal energy, below the planar solutions. An example of a non-planar solution is shown in figure 2*c*. At both ends of the interval, the 3D solutions become planar without any discontinuity.

We can summarize the equilibrium shapes of two filaments with mutually clamped ends as a function of the length ratio *L*_1_/*L*_2_ as follows. At *L*_1_/*L*_2_ = 1, a buckling transition takes place when the force reaches a threshold value. After buckling, the configuration is initially planar. At the length ratio $\Lambda_{1}$, a secondary bifurcation appears and the shapes become non-planar. At the next bifurcation, at length ratio $\Lambda_{2}$, the solution becomes planar again, but with a loop in filament 1. From the equilibrium solution, we can write the parallel and perpendicular component of the force in the joints in the dimensionless form

**Figure 3.**
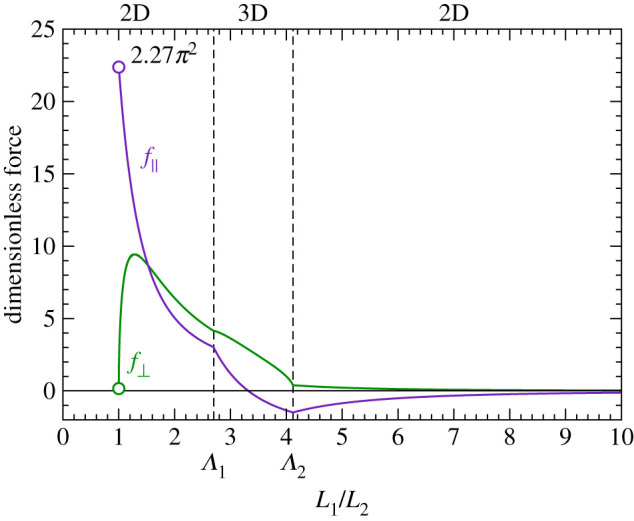
Dimensionless force between the clamped filaments as a function of the length ratio *L*_1_/*L*_2_, as defined in equation (2.14). *f*_║_ denotes the parallel and *f*_┴_ the perpendicular force component.

2.14 $$f_{∥}\left(\frac{L_{1}}{L_{2}}\right)=\frac{L_{2}^{2}}{EI}F\cos\phi_{E}\;\mathrm{and}\;f_{⊥}\left(\frac{L_{1}}{L_{2}}\right)=\frac{L_{2}^{2}}{EI}F\sin\phi_{E}$$

shown in figure 3, which can be used to discuss the active system.

### 2.4. Experimental realization

**Figure 4.**
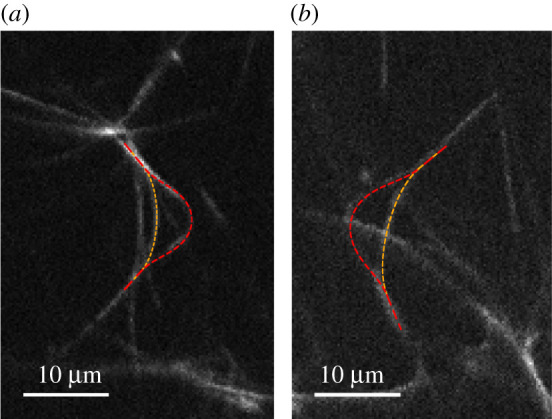
Experimental realization of a microtubule pair and dynein motors (two examples). The shapes show good agreement with the model fit, obtained with parameters: (*a*) *L*_1_/*L*_2_ = 1.36, *ϕ*_*E*_ = 0.70 and (*b*) *L*_1_/*L*_2_ = 1.27, *ϕ*_*E*_ = 0.64.

The planar static shapes predicted by our model can be compared with experimental observations. We carried out experiments by using active microtubule–motor protein systems, which bend under the action of the motors. The experimental set-up was similar to the one reported in [25], but carried out on longer microtubules what allowed stable deformed configurations. Briefly, two fluorescently labelled microtubules were grown from the same seed, i.e. with the same polarity. They were randomly decorated with axonemal dyneins, which collectively self-assembled on one of the microtubules in patches. Under this configuration, the dyneins form cross-bridges to the other filament and buckle it as they walk towards the seed, as illustrated in figure 1. In [25], we observed that the filaments oscillate persistently under these conditions as long as the system is fed with energy from ATP hydrolysis. However, in some cases, the motors shear the filaments until they stall in a strongly curved and planar state. Two examples of such static shapes are shown in figure 4. We tracked the filament contours and we verified that they match perfectly with the fitted curves as predicted by the theory. The agreement suggests that the microtubules are well described with the linear elastica model in the relevant range of curvatures. None of the observed filament pairs entered a non-planar shape, possibly because the dynein patch sizes were in a range where unzipping prevented the build up of a sufficient buckling force. A discussion of dynamical regimes is given in the next section. A systematic mapping of static and dynamical states for different sizes and arrangements of dynein patches, possibly with labelled dyneins, remains an outstanding challenge.

## 3. Active system

In our study on the synthoneme [25], we observed persistent oscillations between two microtubules with fixed minus ends and containing a patch of dynein motors on one of the microtubules. Here, our aim is to study the appearance of such oscillations over a broad range of parameters. Our system consists of two filaments (lengths $L_{1}^{M}$ and $L_{2}^{M}$) that are clamped together at one end (referred to as minus end). Filament 1 consists of an empty segment of length *L*_0_, followed by a segment (length $L_{1}^{M}-L_{0}$) carrying motor proteins (figure 1). In contact with filament 2, the motors produce a force that pushes filament 2 towards the minus end and pulls filament 1 towards the plus end. We use a continuum model for the action of motors, described with a force density *f* = *γ*(*v*_0_ − *v*), where *v* is the relative velocity of the two filaments in the overlapping segment. If the longitudinal component of the elastic force is given by $F_{∥}=(EI/L_{2}^{2})f_{∥}(L_{1}/L_{2})$, the velocity follows as:

3.1 $$v=v_{0}-\frac{F_{∥}}{\gamma L_{\mathrm{overlap}}},$$

where $L_{\mathrm{overlap}}=\text{min}(L_{1}^{M}-L_{1},L_{2}^{M}-L_{2})$ is the overlap length between the section of filament 1 containing motors and filament 2 attached to them. The ability of motors to resist normal loads, when the filaments are being pulled apart, is also limited. We model the detachment in a similar way as has previously been done for motors attached to a surface [33]. We propose that the filaments ‘unzip’ with the velocity $v_{\mathrm{unzip}}=(F_{⊥}-F_{⊥}^{M})/\Gamma$, where $F_{⊥}^{M}$ is the maximum normal load that can be sustained by the motors (note that the normal load only acts on the motors in the unzipping region and is therefore independent of the overlap length). The equations of motion for the segment lengths are

3.2 $${\dot{L}}_{1}=v_{\mathrm{unzip}}\;\mathrm{and}\;{\dot{L}}_{2}=v_{\mathrm{unzip}}-v.$$

We first determine the fixed points for which ${\dot{L}}_{1}={\dot{L}}_{2}=0$. They lead to the condition

3.3 $$\frac{EI}{L_{2}^{2}L_{\mathrm{overlap}}}f_{∥}\left(\frac{L_{1}}{L_{2}}\right)=v_{0}\gamma \;\mathrm{and}\;\frac{EI}{L_{2}^{2}}f_{⊥}\left(\frac{L_{1}}{L_{2}}\right)=F_{⊥}^{M},$$

which can be written in non-dimensional form as

**Figure 5.**
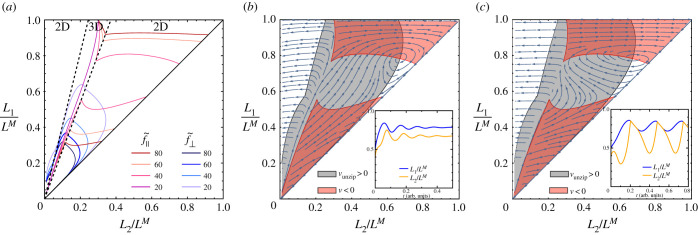
Fixed points and dynamics of the active filament pair. (*a*) Solutions with zero motor velocity *v* = 0 (red) and zero unzipping velocity *v*_unzip_ = 0 (blue), as obtained from equation (3.4). Each curve represents the solution for a value of the dimensionless motor force ${\tilde{f}}_{∥}=v_{0}\gamma {\left(L^{M}\right)}^{3}/EI$ (longitudinal) and ${\tilde{f}}_{⊥}=F_{⊥}^{M}{\left(L^{M}\right)}^{2}/EI$ (transverse). Fixed points of the dynamics are found at the crossings of the two solutions for given parameters. The dashed lines show the borders between planar (2D) and non-planar (3D) shapes. (*b*) The dynamical system for parameters ${\tilde{f}}_{∥}=40$, ${\tilde{f}}_{⊥}=5$ and $\gamma L^{M}/\Gamma =2$. The fixed point is a spiralling sink, indicating that the filaments end in a bent, but stable position. The inset shows the lengths *L*_1_ and *L*_2_ as they approach the fixed points. (*c*) As in (*b*), but with $\gamma L^{M}/\Gamma =0.3$. The fixed point becomes a spiralling source surrounded by a limit cycle. This state corresponds to persistent oscillations (inset).

3.4 $$f_{∥}\left(\frac{L_{1}}{L_{2}}\right)=\frac{L_{2}^{2}L_{\mathrm{overlap}}}{{\left(L_{2}^{M}\right)}^{3}}{\tilde{f}}_{∥}\;\mathrm{and}\;f_{⊥}\left(\frac{L_{1}}{L_{2}}\right)={\left(\frac{L_{2}}{L_{2}^{M}}\right)}^{2}{\tilde{f}}_{⊥},$$

by defining the dimensionless motor forces ${\tilde{f}}_{∥}=v_{0}\gamma {\left(L_{2}^{M}\right)}^{3}/EI$ and ${\tilde{f}}_{⊥}=F_{⊥}^{M}{\left(L_{2}^{M}\right)}^{2}/EI$. In addition, when *L*_1_ = *L*_0_, *v*_unzip_ cannot be negative. *v*_unzip_ = 0 is therefore also fulfilled when *L*_1_ = *L*_0_ and $f_{⊥}(L_{1}/L_{2})<{\left(L_{2}/L_{2}^{M}\right)}^{2}{\tilde{f}}_{⊥}$. A graphical representation of equations (3.4) is shown in figure 5 for the case of equally long filaments $L_{1}^{M}=L_{2}^{M}$. They fall into several distinct regimes: (i) the straight configuration of both filaments is stable if ${\left(L_{0}/L_{2}^{M}\right)}^{2}(1-L_{0}/L_{2}^{M}){\tilde{f}}_{∥}<2.267\pi^{2}$. (ii) A fixed point can exist at *L*_1_ > *L*_0_, see figure 5*b*,*c* for two examples. If the fixed point is stable (figure 5*b*), the stationary state consists of bent filaments and stationary, stalled motors. If, however, it is unstable (figure 5*c*), it is encircled by a limit cycle that represents periodic beating of the filaments. The transition between the two regimes takes place in the form of a Hopf bifurcation. The oscillation frequency at the bifurcation is given by

3.5 $$\omega =\frac{EI}{{\left(L^{M}\right)}^{3}\Gamma }\tilde{\omega }.$$

In the example shown in figure 5, the bifurcation occurs at $\gamma L^{M}/\Gamma =1.05$ with $\tilde{\omega }=40$. (iii) For parameters with no fixed point at all, a limit cycle can still exist that runs via full detachment of all motors. The maximum tip angle during the cycle is then limited to $2\phi_{E}^{\max}=108^{\circ }$. In all the cases discussed above, the necessary condition for generating oscillations is a sufficient filament length without motors *L*_0_ and a sufficient force density to buckle this segment. Note that the derivation holds for a continuum model—if we take into account the stochastic binding and unbinding of motors, random unbinding of motors can possibly replace the motor-free segment.

A quantitative comparison with the experimentally observed shapes shows that the angles can get close to the maximum possible deflection. In the example shown in figure 4*a*, the angle was $2\phi_{E}=80^{\circ }$, 25% below the theoretical limit $2\phi_{E}^{\max}$. We can also estimate the oscillation frequency at the critical point. Using the parameters *EI* = 6 pN μm^2^, *L*^*M*^ = 10 μm and $\Gamma =1\;\mathrm{pN}\;s\;\mu m^{-1}$, equation (3.5) predicts *ω* = 0.24 s^−1^. The oscillations are significantly slower than beating of natural cilia, but similar in frequency to those observed in a related artificial two-filament system [25]. We note that depending on the parameters, the system also allows other oscillatory modes that are not related to the Hopf bifuctation (e.g. the regime (iii) in the above discussion). Their frequency has a more complex dependence on the model parameters.

## 4. Summary

To summarize, we have shown that two filaments with one clamped end and motors acting between them show a variety of static and dynamical regimes. Whether filaments will buckle under load depends on a condition similar to the classical Euler buckling instability, but it differs because the filaments are mutually clamped to each other, rather than to an external support. At higher loads, the filaments can stay arrested in a planar configuration or in one with spontaneous chiral symmetry breaking. When the steady solutions become unstable, cyclic oscillations appear. The oscillations have a non-reciprocal nature, which leads to the conclusion that such filaments could act as a minimal model system for beating cilia and would even generate a net flow of the surrounding fluid. Relatively low beating frequencies are still a deficiency of the simple design—making them faster will likely need additional crosslinking between filaments. Likewise, crosslinking allows the formation of bends and counterbends that lead to flagellar beating. By contrast, our simple system bends the filaments in a single buckled region. Chiral beating patterns are another feature of many cilia [34]. In nature, cilia and flagella exhibit a wide range of beating patterns, ranging from largely planar in respiratory epithelia [35] and microorganisms like *Chlamydomonas* [36] to strongly chiral in ciliates like *Paramecium* or in the vertebrate left–right organizer [37]. The minimal two-filament system we discuss here can also produce both planar and chiral beats, depending on the parameters. A major difference is that the chirality of beats in our system is random and determined by spontaneous symmetry breaking. However, by taking into account that many motor proteins also exert a torque on the filaments they are moving [38,39], even the simple two-filament system could exhibit a defined chirality.

## 5. Material and methods

The experimental set-up was arranged as described in a previous study [25]. Briefly, axonemes were obtained from wild-type *Chlamydomonas reinhardtii* according to the dibucaine method [38,40]. For the extraction of outer dynein arm (ODA) and docking complex from the axonemes, we followed the method described in previous works [27,41]. Demembranated axonemes were resuspended in 0.6 M KCl containing HMDEK solution to extract crude dynein sample and prepare high-salt extract from oda1 axonemes.

The experimental flow chamber was built with Teflon-treated coverslips as previously described in [42] to prevent non-specific binding of proteins onto the surface and spaced with double-sided tape 100 μm thick. Microtubules polymerizing close to each other and with the same polarity were obtained by using fragments of demembranated axonemes prepared by vigorous pipetting and used as seeds attached to the bottom of the flow chamber. After 5 min seed incubation, the experimental chamber was washed with 1% (w/v) Pluronic F127 in BRB80 (80 mM PIPES, 1 mM MgCl_2_, 1 mM EGTA, pH 6.8 with KOH) and incubated for 5 min. Fluorescently labelled (Cy3-labelled) porcine tubulin (3% labelling) was introduced into the flow chamber, polymerized in the presence of 1 mM GTP, 50% DMSO, 1 mM MgCl_2_ at $37^{\circ }C$ for 30 min and stabilized with 7 μM taxol. After microtubule polymerization, diluted crude ODA extract was introduced into the flow chamber and incubated for 5 min. The non-bound protein was eliminated by washing the chamber with buffer and afterwards 1 mM ATP was perfused into the chamber to trigger the activity.

Fluorescence images of the MT-ODA complex were acquired using an inverted fluorescence microscope Ti-E (Nikon, Japan) equipped with a 60 × CFI Apochromat objective (N.A. = 1.49, Nikon, Japan) and the confocal unit (CSU-X1, YOKOGAWA, Japan). The images were acquired at a frequency of 10 Hz. The movement of the filaments over time was tracked manually by using a purpose-written Matlab code. The filament shapes were fitted to the theoretical results by pre-calculating the predicted pair shapes for a dense set of length ratios *L*_1_/*L*_2_, fitting each to the tracked points using a least-squares fitting procedure (GNU Scientific Library (GSL), nmsimplex minimizer) using translation, rotation and scaling as fitting parameters, and finally determining the *L*_1_/*L*_2_ ratio of the best fit.

## Data accessibility

All data are included in the manuscript.

Electronic supplementary material is available online [43].
